# Long-Term-Effects of Training-Accompanied Myofascial Self-Massage on Health Complaints, Symptoms of Overload, and Training Compatibility in Recreational Cyclists

**DOI:** 10.3390/healthcare13111337

**Published:** 2025-06-04

**Authors:** Doris Posch, Markus Antretter, Michael Zach, Martin Faulhaber, Martin Burtscher

**Affiliations:** 1 Department of Sport Science, University of Innsbruck, Fürstenweg 185, A-6020 Innsbruck, Austria; markus.antretter@gmx.at (M.A.); martin.faulhaber@uibk.ac.at (M.F.); martin.burtscher@uibk.ac.at (M.B.); 2 Federal Gymnasium and Federal Realgymnasium, Sillgasse 10, A-6020 Innsbruck, Austria; m.zach@tsn.at

**Keywords:** health complaints, symptoms of overload, training compatibility, myofascial self-massage, foam rolling, Blackroll^®^, thoracolumbar fascia, cycling

## Abstract

**Background/Objectives:** Cycling has become a popular recreational sport, but it can lead to injuries and overload syndromes. The goal of this study is to evaluate the effectiveness of a training-accompanied myofascial self-massage intervention on two primary outcomes: injury occurrence and perceived training intensity. **Methods**: To achieve this goal, we conducted a randomized controlled trial (RCT) with 35 cyclists. A difference-in-differences (DiD) regression analysis was employed to analyze the effects of the intervention. **Results**: The DiD analysis revealed, on the one hand, no statistically significant effect of the intervention on the overall injury score. On the other hand, the intervention group showed a significantly smaller increase in perceived training intensity compared to the control group, supporting the hypothesis that myofascial self-massage decreases the perception of training intensity. In one of our strongest models, which estimated the impact of the intervention from baseline to the second post-test, we observed an adjusted R-squared value of 0.89 and an interaction term coefficient of 1.35 at a significance level of *p* < 0.01. This indicates that, on average, the increase in perceived training intensity was 1.35 points higher (on a scale of 0 to 10) in the control group than in the intervention group. **Conclusions**: This study found no evidence to support the effectiveness of a training-accompanied myofascial self-massage in reducing injury levels, but it demonstrated that the intervention may reduce perceived training intensity. Future studies with larger sample sizes and more objective injury tracking methods are needed to further explore these findings and their long-term implications for injury prevention in cycling.

## 1. Introduction

With the increasing popularity of cycling as a recreational sport, health-related aspects have gained in importance in recent years. On the one hand, recreational cycling can have a positive impact on the physical and mental health of cyclists [1], which is one of the primary motives for athletes to engage in recreational sports [2]. On the other hand, cycling involves the risk of acute injuries and chronic overuse syndromes, which should not outweigh the health and fitness benefits [3]. Moreover recreational cyclists are a growing population that trains regularly, but often lacks individual coaching, structured recovery plans or the ability to conduct biomechanical assessments, which is why a variety of studies have indicated that recreational cyclists and less experienced athletes tend to have higher injury rates than professionals and cyclists with more experience [4,5]. Therefore, it is reasonable to study the health impacts of recreational cycling on athletes.

Sports injuries are in most cases not caused by muscle damage but rather by injuries to the connective tissue [6], which is why experts emphasize the importance of training the fascial network as an intrinsic injury prevention mechanism, highlighting its relevance for both, health as well as physical performance [7]. The fascia as a metasystem is the only system that connects and influences all physiological functions of the body [8]. As empirical evidence suggests, the fasciae play a significant role in proprioception and serve as the primary site of interoception [9]. Rather than exerting force directly on their bony insertions, most muscles transmit a substantial part of their force laterally to adjacent muscles and other structures via myofascial force transmission. Moreover, the interconnected fascial network within muscles likely plays a role in regulating and adapting muscle mass through mechanical and biomechanical signaling [10].

Several factors can contribute to a dysfunctional fascial system, including a lack of movement, poor posture, imbalanced loading, incorrect or excessive strain or overly intense training, which can ultimately lead to muscle pain, tension or movement restrictions [11]. Within the context of cycling, the long fascial line of the posterior chain can experience uneven stress: the calves, thighs and hips typically operate in small ranges of motions for extended periods, often leading to shortening of the hamstrings, while the entire back remains continuously stretched due to the forward-leaning seated position on the bike. Furthermore, structures above the knee and at the hip often show reduced mobility. Unevenly trained structures can also lead to fluid retention in the muscular connective tissue, which may restrict the entire muscle–fascia unit—a problem that can shorten the muscle fibers [12].

Given these characteristics and the implications for the connective tissue, it is reasonable to integrate specific fascial training into personal and athletic training routines. According to Schleip and Müller, the goals of fascia training include the stimulation and activation of fibroblasts to dissolve cross-links, adhesions or myofascial trigger points, thereby restoring the original wave-like structure with a crisscross fiber arrangement, which is often compromised by aging or inactivity [7]. Their study includes a theoretical overview of the main characteristics of the fascial connective tissue and a detailed description of practical exercises, including counter movements, dynamic stretching and myofascial self-massage using foam rollers to release the myofascia. However, the study focused on the theoretical aspects of the fascial connective tissue and did not include an empirical analysis of the effect strength of interventions on injury levels.

Short, regular and long-term (six months to two years) fascia training has been shown to improve movement patterns and coordination, which results in more efficient muscle function, enhanced body awareness, injury prevention, faster recovery times or an improved overall performance [7,12]. In many cases, stretching, massage or other non-invasive rehabilitation tools and techniques are not enough to relax the ITB [13]. To achieve these benefits, athletes can apply various release techniques, such as myofascial release or self-myofascial release using foam rollers [14]. Foam rollers are used for self-myofascial release, which is supposed to be an effective intervention for preventing injuries and overload syndromes, such as the iliotibial band (ITB) friction syndrome [13]. Park et al. found some empirical evidence that foam rollers are an effective technique to release pain and to improve motor performance among cyclists, who were diagnosed with ITBFS [1]. Similarly, Khan et al. concluded in their study on the impact of self-myofascial release on lower back pain that foam rollers are effective in improving hamstring flexibility and balance and decreasing disability levels regarding pain [15].

However, these studies also have their limitations. In [13], the researchers conducted an in vitro analysis of the ITB using human cadavers to examine the effects of stretching. While informative, the results are difficult to generalize within the context of in vivo settings, where biological mechanisms can behave in different ways. Schleip’s edited volume [12], which explores a wide range of topics related to the role of the fascia in sports, provides valuable theoretical insights, but provides limited empirical evidence, especially regarding the effectiveness of foam rollers in reducing injuries and overuse syndromes. Lederman, who focuses on stretching as a strategy to prevent fascia-related injuries and overuse syndromes, points out that *“*stretching before and after exercise has shown no benefit in relieving muscle soreness, it offers no protection against sports injuries, and intense stretching before exercise may even impair athletic performance*”* [16]. Similar inconsistencies and contradictory results can be observed in the research on foam rollers. While Morales-Artacho et al. reported evidence suggesting a causal link between the application of foam rollers and a reduction in muscle stiffness [17], De Camargo et al. found no significant impact of foam rollers on muscle performance or the pressure–pain threshold. The authors speculated that these discrepancies may be due to differences in protocol implementations, especially regarding the duration of the myofascial release exercises [18].

Ilyas et al. analyzed the impact of foam rollers on iliotibial band syndrome (IBS) and concluded that foam rollers reduce IBS. However, their sample size was very small with only 14 participants. Moreover, the study tested two different interventions, one of which used foam rollers and the other one cross fictional massage but did not compare the results with a control group with no intervention at all [19]. Similarly, the study by Khan compared two intervention groups, but did not include a control group without an intervention [15].

In addition to injury prevention and reduction, foam rollers are often associated with factors related to athletic performance and training intensity. For instance, the application of self-myofascial release techniques correlates with an increased range in motion and higher flexibility [20,21,22]. Other studies have shown empirical evidence that self-myofascial massage might reduce muscle fatigue and, therefore, enhance athletic performance during training. It is theorized that self-myofascial release increase muscle blood volume, which might help to remove lactic acids [23]. With its role in reducing muscle fatigue, self-myofascial release may contribute to the perception of athletes that they can work out harder and longer [24]. Michalak et al. found evidence that foam rollers may have an impact on reducing exertion and fatigue [25].

However, these studies also face several limitations and are often difficult to compare due to variations in research design. Many studies have focused on different sport disciplines, tested different types of foam rollers as the intervention or followed varying implementation and measurement protocols. Methodological weaknesses are also common. For instance, studies that rely solely on standard ANOVA to analyze group differences often fail to account for additional variables, which makes it difficult to isolate the true effect of the intervention. Another frequent problem are small sample sizes, potentially leading to Type II errors, in which the analysis fails to detect a real effect, or Type I errors, in which the analysis indicates an effect that does not actually exist.

The following study addresses a variety of these issues and analyzes the potential impact of training-accompanied myofascial self-massage on health, injuries and overload syndromes, on the one hand, and on the perceived training intensity, on the other. To achieve this goal, the authors conducted an exploratory longitudinal randomized controlled trial (RCT) analyzing the long-term effects of a six-month intervention involving training-accompanied myofascial self-massage in the lower extremities and the *Fascia thoracolumbalis.* For the myofascial self-massage, the Blackroll^®^ was applied twice a week, immediately following a cycling training session.

The present study differs from the cited studies in its longer intervention period. There is no control group with a different intervention. The longer use of the foam roller, which includes a comprehensive training program for all fascial structures involved in cycling, is one of the key differences. In contrast to ANOVA, this study controls the additional variables and analyzes causal influence using the interaction term.

The analysis of the training-accompanied myofascial self-massage in the following study focuses on two primary goals: (1) the impact on the frequency of health complaints and overuse symptoms and (2) the impact on training tolerance and perceived training intensity. To answer these questions, this study uses a difference in differences (DiD) regression analysis [26], which allows us not only to measure the quantitative causal effect of the intervention on the injury level and the perceived training, but also to control for additional variables. According to our questions, we formulated five hypotheses:

- **H_1_:** *The application of training-accompanied myofascial self-massage leads to a reduction in health complaints and overuse syndromes related to cycling*.
- **H_2_:** *The application of the training-accompanied myofascial self-massage results in a decreasing perception of training intensity*.
- **H_3_:** *There are correlations between age and the target variables*.
- **H_4_:** *There are correlations between BMI and weight and the target variables*.
- **H_5_:** *There are correlations between the type of bicycle used by participants and the target variables*.

## 2. Theoretical Background and Hypothesis

This study investigates training-accompanied myofascial self-massage using the Blackroll^®^ as the independent variable and its impact on various dependent variables. The Blackroll^®^ is a foam roller used in various exercises to increase the elasticity and performance of the muscles and the fascial tissue [27]. During the trial preparation phase, the participants received detailed instructions on how to apply the Blackroll^®^. With the beginning of the intervention phase, the participants were required to follow a specific training program consisting of twelve exercises, which target different body parts and tissues, as recommended by the manufacturer. These exercises focused on the plantar fascia, calf, tibialis anterior, quadriceps, hamstring, adductors, iliotibial band, psoas, gluteal muscle, lower back as well as upper back [28,29]. The application of the Blackroll^®^ was performed twice a week immediately after a cycling training session for a duration of six months. The participants were not allowed to conduct additional accompanying measures such as massages or other self-massages.

Whilst the intervention serves as the main independent variable, we will also test two different main dependent variables. The first main dependent variable will be the overall level of injuries and overload syndromes, which is quantified using a composite injury score that incorporates the number, frequency, timing and intensity of reported injuries (for details, see Section 3.3, “Operationalization of the variables”). Thus, the first hypothesis builds on the empirical studies that have examined the relationship between the application of a foam roller and injury occurrence (see [13,16]) as well as the theoretical research that discusses the potential health benefits of foam rolling as a myofascial release technique (see [7]). To analyze the impact of the intervention on the overall level of injuries and overload syndromes, this will be tested with the following main hypothesis:

**H_1_**: *The application of training-accompanied myofascial self-massage leads to a reduction in health complaints and overuse syndromes related to cycling*.

While the hypothesis H_1_ investigates the level of injuries and overuse syndromes, the second hypothesis focuses on the subjective perception of training intensity, which is measured using the 10-point rate of perceived exertion (RPE) scale, a validated tool in sports science [30]. Based on the findings of various studies [20,21,23,24], which we discussed in the previous section, we theorize that the intervention results in a decreasing perception of the training intensity. To test the impact of the intervention on the perceived training intensity, we formulated the following hypothesis:

**H_2_**: *The application of the training-accompanied myofascial self-massage results in a decreasing perception of training intensity.*

In addition to the intervention as the primary independent variable, the analysis will test a range of hypotheses that account for the additional control variables that may influence the target variables. One of these variables is the age of the participants. Numerous studies suggest that the variable *age* may influence both the frequency and type of injuries and overuse syndromes. For instance, DeHaven and Lintner found that while some injuries, such as knee injuries, are common among all age groups, other injuries, such as inflammation or traumatic arthritis, occur more frequently in older individuals [31]. Similarly, Stevenson et al., who analyzed injury rates among recreational athletes from different sports disciplines, found statistically significant evidence that older participants were more likely to be injured than younger ones. Specifically, individuals aged 26 to 30 had a 55% greater risk of injury compared to athletes under the age of 18 [32]. Age-related effects have also been studied within the context of perceived training intensity and exertion [33]. Since various studies observed differences in injury rates based on the variable *age*, we collected age data via a demographic questionnaire and treated it as a continuous covariate in the analysis. To test the variable age, we formulated the following hypothesis:

**H_3_**: *There are correlations between age and the target variables*.

Another factor, which is frequently examined as a variable influencing the frequency and type of injuries, is the variable BMI. A study by Tyler et al., which investigated risk factors for ankle injuries among high school American football players, found that BMI was a statistically significant predictor of injury occurrence [34]. Similarly Anam et al. reported that athletes with a higher BMI experienced higher injury rates compared to those with a lower BMI [35]. Several theoretical explanations are suggested by the authors of the studies. One explanation is that a higher BMI can increase the mechanical load on joints, such as the ankles, potentially exceeding their structural stability during training. Additionally, elevated BMI is often associated with lower levels of overall fitness, including reduced strength, endurance, balance and coordination, all of which may contribute to a greater risk of injury [36]. Anam et al. speculated that a higher BMI may limit the ability to move or influence the shape of body parts, such as the foot shape, which could cause higher risk of injury [35]. In regard to our second main dependent variable, Salvadego et al. found evidence among cyclists that obesity increases O_2_ costs and, therefore, increases the perceived exertion [37]. BMI was calculated based on self-reported height and weight from the questionnaire, using the standard formula: weight in kilograms divided by height in meters squared.

**H_4_**: *There are correlations between BMI and weight and the target variables*.

Finally, in our last hypothesis, we test the relationship between the type of bicycle used by participants and the target variables *overall injury level* and *perceived training intensity*. A variety of studies have indicated that the frequency and type of injuries can be affected by the specific characteristics of different sports disciplines [31]. Similarly, Stevenson et al. found differences in the frequency and type of injuries between different sports disciplines. According to their findings, disciplines with less body contact, such as netball, tend to have lower injury occurrences than disciplines with more body contact, such as football or soccer [32]. Although there is a lack of epidemiological studies examining and comparing injury occurrence across different cycling disciplines, we assume that different cycling disciplines may also be associated with distinct injury profiles. Cycling is a diverse sport with various disciplines and subdisciplines, each of which involves different physical demands, environmental conditions and risk factors [38].

**H_5_**: *There are correlations between the type of bicycle used by participants and the target variables*.

To measure and operationalize the type of bicycle, participants reported their annual distribution of bicycle use across categories—road, mountain, indoor or other—as percentages. For a detailed description of how the variables were quantified and operationalized, see Section 3.3 “Operationalization of the variables”.

## 3. Study Design and Data

### 3.1. Study Design

To examine the impact of the Blackroll^®^ on the health of cyclists and overuse syndromes, this study employed an exploratory, longitudinal randomized controlled trial (RCT). RCTs are widely considered the gold standard for assessing the causal effects of an intervention or a treatment [39]. The intervention in the following analysis involved a regular training-accompanied myofascial self-massage of the fasciae using the Blackroll^®^ twice a week in the lower extremities and the Fascia thoracolumbalis immediately after cycling training. The participants were randomly divided into an intervention group, which integrated the Blackroll^®^ into their training program, and a control group, which participated in the trial and continued regular cycling training but without using the Blackroll^®^.

Another key feature of RCTs is that data is collected at multiple points in time [40]. According to the scholarly literature, fascial structures adapt slowly. To achieve positive and lasting effects, regular long-term fascial training—over a period of six months to two years—is recommended, which involves a short exercise program lasting only a few minutes per session. The fibroblasts stimulated in the connective tissue initially increase their collagen degradation activity within the first one to two days, after which collagen synthesis becomes predominant. Therefore, fascial training should ideally be performed once or twice per week [41]. For this study, the intervention phase lasted six months, in which this study began with a baseline measurement prior to the intervention, followed by two follow-up measurements during and after the intervention period (first post-test, second post-test). The first post-test took place three months after the trial had started and the second post-test three months after the first post-test.

### 3.2. Data Collection

This study focuses on adult recreational cyclists as the target population. The participants were selected by the authors based on a number of inclusion and exclusion criteria. Despite extensive efforts, only three female cyclists could be recruited, so this study was ultimately conducted with only male cyclists. Originally, we recruited 36 cyclists; however, one participant dropped out during the trial. Therefore, the total sample size (N) consisted of 35 male recreational cyclists, who were divided into two groups, 18 in the intervention group and 17 in the control group. Based on the results of the VO2max determined during the baseline tests, participants were assigned to the intervention or control group in descending order in blocks of four. All participants had a comparable baseline level, with an average training volume of 8 to 10 h per week and a minimum of three years of cycling-specific training experience. This ensured that participants’ cycling-specific starting levels were as homogeneous as possible. The scope and intensity of the intervention corresponded to the same physical and psychological demands of a trained recreational cyclist. This ensured that any increase or decrease in training load would not distort the results. The age range of participants was between 26 and 56 years at the time of the trial to additionally investigate differences between different age groups. This study was conducted in cooperation with all 35 registered cycling clubs across Tyrol, Austria. Recruitment was carried out through written emails distributed to all cycling clubs and lasted from mid-March to mid-September. We used a non-probabilistic sampling procedure in the form of consecutive sampling. This involved selecting participants who met all our typical selection criteria for recreational cyclists during the recruitment phase of this study. Participants were therefore selected based on the researchers’ expertise, assuming that the selected samples were free of bias and representative of the reference population. The researchers assume that road cyclists who participate in amateur cycling several times a week in a cycling club have relatively similar characteristics. In terms of performance, they are likely all significantly above the average for the general population, but below the average for professional road cyclists. Furthermore, injury patterns (spine and knee) are similar, which supports the assumption that road cyclists are relatively well represented.

To be eligible for this study, participants had to meet the following criteria:

- Age between 20 and 56 years at the time of the baseline test;
- Active recreational cyclist;
- Average training volume of 8 to 10 h per week;
- At least three years of cycling-specific training experience;
- A spiroergometric test performed within the last two years;
- Pulse-controlled training for the past three years;
- No training interruptions longer than six weeks in the past three years;
- A medical health certificate, issued within the last two months, confirming no contraindications or risks associated with maximal exertion until exhaustion.

In addition to the inclusion criteria, participants were excluded from this study if they met any of the following conditions:

- Licensed competitive cyclists;
- Individuals diagnosed with osteoporosis, disc damage, thrombosis, high blood pressure (above a specific threshold), fibromyalgia or soft tissue rheumatism;
- Individuals with a knee or hip joint implant;
- Participants who developed acute or chronic illnesses during this study.

Participants were excluded from the analysis if they dropped out, as no intent-to-treat analysis was conducted. The discontinuation criteria were categorized as follows:

- Study dropouts: Individuals, who voluntarily withdrew from this study, were not included in the evaluation;
- Intervention-related events: This included adverse events, intercurrent illnesses or failure to adhere to the training protocol;
- Non-intervention-related events: This included withdrawal of consent or the discovery of exclusion criteria after this study had begun.

The intervention group followed a structured, precisely defined, pulse-controlled 80 min cycling training session twice per week. Training was conducted in the G1 and G1/G2 intensity zones (aerobic–anaerobic transition zone with lactate levels between 2 and 4 mmol/L) and included strength–endurance intervals. Additionally, they performed a standardized myofascial self-massage of the fasciae of the lower and upper extremities and the thoracolumbar fascia using the Blackroll^®^, immediately after each cycling session. The control group was instructed to follow the exact same cycling training as the intervention group, but without applying the Blackroll^®^.

During the tests, the training and measurements followed a highly controlled protocol. First of all, the tests were conducted on the participants’ own race bicycles, starting with 100 watts and a 50 watt increase every three minutes. Moreover, the training was heart rate-controlled and took place indoors. The spiroergometric and lactate measurements were conducted with the CYCLUS 2. Spiroergometric and lactate measurement using the CYCLUS 2 on the participants’ own racing bike allowed us in this context to determine heart rate zones for training control. The aerobic and anaerobic thresholds were determined according to Mader A. at 2 and 4 mmol/l. During the tests, we independently measured power meter pedals with the Vector2™, along with a compatible Edge^®^ bike computer, both of which were obtained from Garmin^®^. This meant that both pedals were equipped with measurement electronics, which allowed us to measure the power output and force distribution separately for each leg.

Both groups completed a precisely structured, 80 min heart rate-controlled cycling session twice per week in the aerobic zone (basic endurance 1, GA1) and (basic endurance 2, GA2), with lactate levels between 2 and 4 mmol/l at a cadence of 90 to 100 revolutions per minute (RPM). After a 10 min warm-up in the lower GA1 range, the session continued with 3 to 5 min extensive strength endurance intervals (KA intervals) in the GA2 range at 60 to 70 RPM. These intervals were performed with a 1:1 ratio of interval to recovery, with the recovery taking place in the GA1 range at 90 to 100 RPM.

Additionally, we implemented the training based on a so-called block periodization with a total number of six blocks, each of which lasting four weeks. After each block training, the intensity was increased by 5%, based on the individual performance at 2 mmol/L determined during the baseline test. KA intervals began in the first block in the lower GA2 range and ended with increasing intensity after six months in the upper GA2 range. Table 1 presents a detailed overview of the structure of the training blocks.

Each training session was fully recorded, including the heart rate, wattage and cadence, and subsequently documented in terms of training intensity to ensure that all parameters were strictly followed. For monitoring purposes, participants kept a training diary throughout the entire intervention period, in which all endurance sports activities, including non-cycling-related training activities, were precisely documented in terms of duration (5 to 7 h per week) and intensity (in the GA1 range).

This study also utilized block randomization. A randomization list was managed by the project leadership, who assigned participants to either the intervention or control group based on this list once the author of this study obtained their informed consent.

The intervention phase followed a structured plan for recreational cyclists designed by the author, based on many years of experience as a state-certified trainer, a teaching qualification for movement and sport and former successful cyclist at national and international level. Before this study began, a three-week preparatory phase ensured that participants received detailed personal instructions on how to properly execute the training protocol. Furthermore, the author conducted pretests prior to the intervention phase to validate and test the data collection process and prevent potential measurement errors. The pretest involved twelve amateur cyclists within the context of a seminar, during which we tested our setup and measurement protocols. The recruitment of the participants, instructions and pretests took place in September (week 37–40), followed by the baseline test in early October (week 41–42). The intervention began mid-October (week 43) and lasted 24 weeks, during which participants completed two precisely defined, pulse-controlled cycling training sessions per week, each lasting 80 min within the aerobic-anaerobic transition zone, at intensities that result in a lactate concentration of 2 to 4 mmol/l. Both the intervention and control groups followed these trainings with strength-endurance intervals of four weeks each. After three months, we conducted the first post-test and after six months the second post-test. In addition, a food diary was kept throughout the entire period (see Supplementary Materials Table S1).

### 3.3. Operationalization of the Variables

The health complaints and overload syndromes, which represent the dependent variables in H_1_, were measured by a standardized questionnaire, assessing the subjective perception of various health complaints and overload syndromes. The questionnaire was designed by the authors, validated by two faculty members and tested on twelve cyclists as part of the pretests. The cyclists, who helped to validate the questionnaire, were not part of the actual trial. Based on their feed during the pretest, minor linguistic adjustments were made to improve clarity. The final version of the questionnaire was then re-evaluated by the authors and reviewed by two faculty members. The possible answers in the questionnaire regarding location, type, frequency, timing and intensity of the complaint were precisely defined. Participants completed the questionnaire anonymously by hand after the spiroergometry during the baseline test, first post-test as well as the second post-test.

The questions and attributes used to assess the complaints and overuse syndromes were partially derived from Tofaute [42], a study conducted by the Cologne Sports University. Furthermore, the conceptualization of this project was combined with survey designs developed by Kromer et al. [43] and Froböse et al. [44]. The same questionnaire was used for all tests, including the baseline test before the intervention phase, the first post-test and the second post-test, which marked the end of the trial for the participants. The assessment of the health complaints and overuse syndromes consists of various sections, including questions on (1) the localization and frequency of the complaint, (2) the type of the complaint, (3) the point in time of the complaint and (4) the intensity of the complaint. The various health complaints and overuse syndromes were assessed for different localizations and body parts, including finger and finger joints, wrists, arms, shoulders, neck and cervical spine, lower back and lumbar spine, gluteal, hip joint, upper legs, knee, feet and toes or other complaints. To assess the frequency of complaints, participants were able to choose between *rarely* and *frequently*. No response indicated that the participant did not experience any health complaints or overload injuries. In addition to the localization of the complaint, the questionnaire asked for the type of complaint, including *pain, tensions, convulsion, formication, numbness, stiffness* and *skin problems*. Regarding the timing of the complaint, the survey asked whether the complaint occurred *during* or *after the cycling training* or *both*. The question about the intensity of the complaint included three attributes: *tolerable, disturbing* and *intolerable*.

For the statistical analysis of H_1_, which tests the overall level of complaints, injuries and overload syndromes, we calculated a factor variable based on a cumulative score, which combines all body parts and attributes, consisting of (1) the total number of complaints (1 point per injury or complaint, 2 points for two complaints, etc.), (2) the frequency of complaints (0 point for no occurrences, 1 point for rare occurrences and 2 points for frequent occurrences), (3) the timing of the complaint (0 point for no complaints at all, 1 point for during the training, 1 point for after the training and 2 points for both) and (4) the intensity of the complaint (1 point for tolerable, 2 points for disturbing and 3 points for intolerable). In sum, we calculated a metric variable with a score that combines different factors and represents the overall level of injuries and complaints. Scores were calculated for each specific body part, with higher scores indicating more severe injuries and complaints.

H_2_ examines the impact of the application of the Blackroll^®^ on the perception of the training intensity. Perceived training intensity was measured using the 10-point version of the RPE scale, ranging from 0 (nothing at all) to 10 (extremely strong), which has shown reliability and validity especially when used among healthy, clinical and athletic adult populations [30]. Finally, H_3_-H_5_ investigate the correlation between generic physical indicators as independent control variables, on the one hand, and on the other, the level of injuries and the perceived training intensity as dependent variables. The generic physical indicators included questions about the age, body weight and body height. Finally, the survey collected generic data related to the cycling training, such as the type of bike used by participants and their annual relative distribution or whether and how often participants engaged in compensatory training.

### 3.4. Data and Structure of Dataset

This leads us to the characteristics of the data and the structure of the dataset. The questionnaires and measurements were conducted during the baseline test, first post-test and second post-test. For the final dataset, one participant was excluded for violating the inclusion criteria. As a result, the dataset includes data for 35 recreational cyclists, of whom 18 were in the intervention group and 17 in the control group. Due to the measurements and the structure of the questionnaire, the final dataset contained 244 different variables.

The first cluster of variables provides physical and demographic data, such as age group, body weight, body height, BMI or group assignment (intervention or control group). Another set of variables includes data on cycle training, such as different types of bicycles and their relative distribution in percentages or the frequency of compensatory training. A third cluster of variables focuses on injuries and complaints affecting different body parts, including fingers, wrists, arms, shoulders, neck and cervical spine, lower back and lumbar spine, gluteal, hip joint, upper legs, knee, feet and toes as well as other complaints. For each body part, the dataset provides information on the frequency of complaints, type of complaint, intensity of the complaint and whether the complaint occurred during and after the cycling or both. For the second main dependent variable, the dataset includes an ordinal scaled variable between 0 and 10 measuring subjective and perceived training intensity. In addition to cross-sectional data, the dataset includes longitudinal data for each of these clusters, with three data points collected during the baseline test, first post-test and second post-test. Table 2 shows an overview of the most important variables and structure of the dataset.

## 4. Statistical Methods

Before this study analyzed the different hypotheses, the first step of the statistical analysis is to provide a general overview and frequency distributions of the most important variables using methods from descriptive statistics. This includes frequency statistics, averages and graphical representations illustrating the distribution of key variables and their attributes. Part of the descriptive analysis is the characterization of the sample. Another part is a correlation analysis, which investigates the relationships between the variables. The main goal of the correlation analysis is to obtain an overview of bivariate relationships. Another goal of the correlation analysis is to check whether the independent variables correlate with each other to avoid multicollinearity. To conduct the correlation analyses we used Spearman correlations.

In the second step of the statistical analysis, we used methods from inferential statistics. One of the most important goals of randomized controlled studies with an intervention group and a control group, measured at multiple points in time, is to analyze the causal effects of an intervention using statistical methods. One such method used for analyzing causal impacts is the difference-in-differences (DiD) regression model, which has become a popular approach for intervention analysis [45]. DiD analysis can be applied in cases where the outcome of a specific intervention in a treatment or intervention group is analyzed and compared with a control group both before and after a treatment or intervention takes place [46]. In a simple DiD regression analysis, we controlled the time and group and focused on the interaction term, which includes both time and group. The two differences in the DiD model represent, on the one hand, the difference in the means of the response variable between the intervention group and the control group after the treatment and, on the other hand, the difference in the means between the intervention group and the control group before the treatment [43]. By calculating the difference-in-differences, we obtained the so-called treatment effect or DiD estimator δ, which can be expressed by the following formula:

(1) $$\hat{\delta }=\left({\bar{y}}_{T,A}-{\bar{y}}_{C,A}\right)-\left({\bar{y}}_{T,B}-{\bar{y}}_{C,B}\right)$$

where ${\bar{y}}_{T,A}$ represents the mean of the treatment group after the intervention; ${\bar{y}}_{C,A}$ the mean of the control group after the intervention; ${\bar{y}}_{T,B}$ the mean of the intervention group before the intervention and ${\bar{y}}_{C,B}$ the mean of the control group before the intervention. 

In the following analysis, the DiD estimator will be integrated into a DiD regression model, which has the advantage that additional factors or covariates can be integrated and controlled. The DiD regression model consists of indicator variables for the intervention before and after the treatment as well as their interaction term. Note that in the DiD regression model, the coefficient δ on the interaction term exactly represents the DiD estimator in Formula (1) [47]. In its simplest implementation, the DiD regression model can be defined by the regression formula

(2) $$y=\beta_{0}+\beta_{1}T+\beta_{2}A+\delta \left(T\times A\right)+\varepsilon$$

in which y represents the response variable; $\beta_{0}$ is the baseline value before the intervention started and is applied to the control group—in the regression model, the intercept, $\beta_{1}$ captures the effect of time on the outcome variable, which affects both groups equally and T is a time dummy variable with a value of T=0 before the intervention and T=1 after the intervention. A is a treatment group dummy variable with A=0 for the control group and A=1 for the treatment group, whereas $\beta_{2}$ reflects the pre-existing differences between the treatment and control groups before the intervention. The interaction term $\delta \left(T\times A\right)$ represents the treatment effect, measuring the causal impact of the intervention by comparing how the treatment group changes relative to the control group or how many more units one group changed compared to the reference group. The interaction is what makes the DiD regression model different from a simple before–after comparison, as it accounts for any general time trends that affect both groups. Finally, ε represents the error term, which includes all unobserved factors that might influence the response variable.

Since the analysis includes additional variables, which will be tested as covariates, the regression formula can be extended by incorporating control variables $X_{1}$ and $X_{2}$ and their respective coefficients $\beta_{3}$ and $\beta_{4}$:

(3) $$y=\beta_{0}+\beta_{1}T+\beta_{2}A+\delta \left(T\times A\right)+\beta_{3}X_{1}+\beta_{4}X_{2}+\ldots +\varepsilon$$

A specific feature of general DiD models is that they only consist of the two time periods before and after. Since the data in the following analysis includes three time periods, the DiD regression analysis in this study requires a specific two-part procedure. In the first part, conventional DiD regression models with two time periods will be conducted, using the DiD regression Formulas (2) and (3). This includes the following:

- Differences between the baseline test (before the start of the intervention) and the second post-test (end of the trial);
- Differences between the baseline test (before the start of the intervention) and the first post-test (end of the first intervention phase);
- Differences between the first post-test (end of the first intervention phase) and the second post-test (end of the trial).

Comparing the individual DiD regression models provides the opportunity for a more fine-grained analysis of the progress during the trial. In the second part, a dynamic regression model will be tested, incorporating a baseline test and two post-tests. DiD regression models, which extend beyond the conventional 2 × 2 setting, include separate treatment effects for each post-intervention period, which can be expressed by the following regression formula:

(4) $$y=\beta_{0}+\beta_{1}T+\beta_{2}A+\sum_{h=0}^{H}\delta_{h}D_{t*+h}\times A+\sum_{k=1}^{K}\beta_{k}X_{kit}+\varepsilon$$

where y represents the response variable; $\beta_{0}$ is the intercept of the regression line with the y-axis, which is in this case the baseline outcome; $\beta_{1}T$ reflects the time effect, which captures the trends over time and $\beta_{2}A$ is the group effect, which represents the baseline differences between the intervention and control groups. The term $\sum_{h=0}^{H}\delta_{h}D_{t*+h}\times A$ defines the dynamic treatment effects, in which the $D_{t*+h}$ term are dummy variables for different post-treatment periods and $\delta_{h}$ stands for the treatment effect at different points in time. The part of the formula in $\sum_{k=1}^{K}\beta_{k}X_{kit}$ represents the additional control variables and their coefficients and the ε is the error term that accounts for the influence of other factors. The regression models were derived and adjusted based on the descriptions in [45,46,47]. SPSS 25.0 and Python 3.9 were used to perform the statistical analyses.

In addition to analyzing the effects of the intervention, this study incorporated several control variables in the regression models to account for potential influences on the dependent variables. The inclusion of these covariates was mainly driven by theoretical considerations based on the previous research linking age and BMI to injury risk and perceived training intensity, as well as assumptions about variation in injury patterns across cycling disciplines. The variables were selected because they are commonly reported in the literature as relevant risk factors for physical complaints and exertion. Moreover, the variables were available through the questionnaires completed by each participant.

In addition to the theoretical considerations, we conducted a correlation analysis between all independent and dependent variables for variable selection. When two potential predictors were correlated with each other, we dropped the variable with a lower coefficient and/or lower significance to avoid multicollinearity, by which we made sure that our models are theoretically sound and statistically robust.

To further assess the robustness of the models and evaluate the contribution of the control variables, we compared nested regression models with and without these covariates. The primary model included only the intervention as the independent variable; meanwhile, in the extended model, we added age, BMI and bicycle type. Model comparisons were based on changes in R^2^ and adjusted R^2^ values and F-tests for nested models where appropriate. The inclusion of the control variables slightly improved the model fit in most cases, which suggests that they account for some of the variance in the dependent variables, although not to an extent where it changed the direction or significance of the primary intervention effects. The selection of the final regression model in our study followed a nuanced, multi-step process. We compared coefficients, significance levels, *t*-tests and R-squared values across different model specifications to identify the most meaningful and robust results.

The final step in evaluating the robustness of the models was the calculation of corrected *p*-values using the Holm–Bonferroni method. The Holm–Bonferroni method is a commonly used approach to control family wise errors (FWER). According to the statistical literature, the Holm–Bonferroni method controls the FWER, while rejecting more null-hypotheses than the classic Bonferroni correction. As a result, it reduces the risk of Type II errors and thus provides more statistical power. The Holm–Bonferroni correction does not make any independence assumptions and is at least as strict as the classic Bonferroni method [48]. Moreover, we conducted a Shapiro–Wilk test to assess whether the residuals are normally distributed, which is a key assumption of linear regression.

## 5. Results

In the following section, we present the experimental results, their interpretation and the conclusions derived from the data, which consists of two subsections. In the first subsection, we provide a general overview of the sample and the most important variables, using methods from descriptive statistics. In the second subsection, we analyze the hypotheses based on the DiD regression models.

### 5.1. Descriptive Analysis of the Participants’ Basic Physical Characteristics

The descriptive analysis of the sample reveals the basic characteristics of the participants and the main variables. The data appears to be skewed toward the middle age groups, with the highest frequency occurring in the 41–50 category (18 participants), followed by the 31–40 category (11 participants). The 20–30 (one participant) and 51–60 age groups (five participants) have significantly lower frequencies, indicating that most participants fall within the middle age ranges. This suggests that the sample is not evenly distributed across age groups and is concentrated in the 31–50 range. The most notable observation is that both groups have the same peak in the 41–50 age category. However, there are some differences in the distribution of the categories. The intervention group has a higher frequency in the 31–40 category compared to the control group, suggesting that slightly younger participants were more likely to be assigned to choose the intervention. In contrast, the control group is more evenly spread across the older categories, particularly in the 51–60 range. The 20–30 age category has the lowest representation in both groups, which means that younger participants are underrepresented (see Supplementary Materials Figure S1).

Several differences can be observed in the distribution of BMI classes for the intervention and control groups. Most participants in both groups fall within the 21–25 BMI range, indicating that most individuals have a normal BMI. However, there is a noticeable peak in the normal weight category for the control group, where its frequency is higher than in the intervention group. In contrast, the intervention group has a higher representation in the overweight class. This suggests that the intervention group includes individuals with a slightly broader range of BMI values, potentially influencing the results if BMI plays a role in the intervention outcomes. The absence of participants in the extreme BMI categories (<19 or >31) in both groups indicates that most individuals fall within a moderate range, which could be beneficial for the comparability of the two groups. In the overall sample, the participants have on average a BMI of 23.85, with a minimum of 19.49, a maximum of 29.7 and a standard deviation of 2.3. Given these differences in both groups and their potential impact on health complaints, it is reasonable to consider the BMI as a control variable in the regression models (see Supplementary Materials Figure S2).

### 5.2. Descriptive Analysis of Health Complaints and Their Frequencies

Before we dive deeper into the analysis and investigate the impact of the intervention on health complaints using DiD regression analysis, the following section presents a descriptive overview of the different health complaints and overuse syndromes for the intervention and control group. Table 3 below shows the share of participants (in % of their group) and the total number of individuals who reported a health complaint for each specific body part at the baseline test (before the intervention started). There are a couple of interesting insights that can be drawn from the descriptive analysis of the number of injuries and complaints, one of which is that a relatively high number of individuals in both groups reported health complaints in relation to their spine. A notable difference between the intervention and control group when it comes to spine problems is that the intervention group shows a high amount of neck/cervical spine problems (44%), while the control group reported a higher number of individuals with lumbar spine complaints (29.4%). In the overall sample, 60% of the participants either reported problems with the neck and cervical spine or the lumbar spine.

Another body part which is associated with a significant number of health complaints is the knee with an overall share of 34.3% of the individuals who reported knee problems before the intervention started. Both groups reported the same total number of complaints regarding the knee. As can be seen in Table 3, spine and knee problems are followed by complaints regarding the fingers (seven complaints), the wrists (seven), upper legs (six), gluteal (five), shoulders (five), hip joint (four), feet/toes (four), other complaints (four) and arms (two). Finally, it should be mentioned that both groups showed a similar number of injuries, with a total number of 40 complaints in the intervention group and 37 complaints in the control group.

### 5.3. Descriptive Analysis of the Overall Score of Complaints and Injuries

The following section includes a descriptive analysis of the scores that represent the overall level of injury for each body part. In contrast to Table 3, which contains the total number of injuries and complaints at the baseline test, the scores in Table 4 are calculated based on the frequency and intensity of the injury for each body part. Like the ranking in Table 3, the highest score occurred for the neck/cervical spine in the intervention group with a total score of 45, followed by knee injuries with a total score of 33 in the control group and a score of 32 in the intervention group. Although most body parts show a similar level of injuries in both groups, there are a few body parts with major differences between the two groups, one of which is the neck/cervical spine. Another major difference between the two groups can be observed at the hip joints with a score of 21 in the intervention group and a score of 3 in the control group.

Whereas in most cases the ranking of the scores correlates with the absolute number of injuries, the gluteal complaints rank higher in Table 4 than in Table 3, which could mean that the complaints related to the gluteal tend to be more severe and frequent. As can be seen in Table 4, the intervention group showed a higher overall score with 223 points compared to 187 points in the control group, which can be partially explained by the fact that the intervention group had a slightly higher total number of injuries and complaints.

Table 4 shows, on the one hand, the scores for each body part and each group in absolute terms at the first post-test and the second post-test and, on the other hand, the changes in the score compared to the previous test. As can be seen in the table below, the biggest change occurred for the neck/cervical spine in the intervention group with a total reduction of 30 score points, followed by the lumbar spine in the control group with a decrease of 18 score points. The most significant changes occurred in both groups after three months in the trial (first post-test) with a total reduction of 105 score points in the intervention group and 78 score points in the control group. The intervention group also showed a greater reduction in the overall injury level at the second post-test compared to the control group, even though the changes were significantly lower than the changes at the first post-test. In other words, the intervention group showed a slightly greater reduction in the overall injury level than the control group at the first and second post-test, which could mean that the application of the Blackroll^®^ may have a slight impact. However, the difference in the average change per participant between the intervention and control group is very minimal because the control group also showed a significant reduction in their overall score, which means that other influential factors might have to be considered as well—some of those potential factors will be analyzed as part of the correlation analysis and regression models.

The development of injury scores across the three measurement points reveals that both the intervention and control groups experienced a reduction in injury-related complaints from baseline to the first post-test. The intervention group’s total score declined from 223 to 118, which reflects an average decrease of 5.83 points per participant. Similarly, the control group’s score dropped from 187 to 109, which is an average decrease of 4.6 points per participant. The subsequent changes between the first and second post-test were minimal in both groups: the intervention group showed a slight decline of 1 point in total, or 0.56 points per participant, while the control group improved by 9 points, or 0.53 points per participant. These findings indicate that the most substantial improvement occurred during the initial intervention period and the changes affected both groups, suggesting that general training adaptation, seasonal effects or other factors may have contributed to a reduction in the injury score over time rather than the intervention.

### 5.4. Correlation Analysis of the Main Variables

As part of the correlation analysis, we first investigated the first main variable: the overall injury score, which reflects the number of injuries and complaints and their extent and severity. The overall score shows only one statistically significant correlation in the dataset, which is a moderate, negative correlation with the variable bicycle type, mountain bike (r = −0.37, *p* < 0.05). This may suggest that individuals who primarily use a mountain bike tend to report fewer overall complaints or injuries. Theoretically, it is possible that mountain bikers engage in more varied and technically demanding riding styles that promote physical resilience. Another explanation would be that mountain bikers are less likely to injure their spine due to their position on the bike, which differs from the position of racing bikers. Regarding the anthropometric variables such as BMI, body weight or age, we originally expected a higher BMI or older age to be associated with a higher level of injury and complaints, but the correlation analysis does not support this assumption.

The second main variable is the subjective intensity of the training, for which we can observe several significant correlations. The strongest correlation can be observed between the intensity and body weight (r = 0.71, *p* < 0.01), whereas the BMI (r = 0.37, *p* < 0.05) and height (r = 0.45, *p* < 0.01) show moderate correlation with intensity, indicating that heavier and taller individuals also show higher intensity levels. The strong correlation between intensity and body weight suggests that individuals who weigh more may perceive their training more intensively. Contrary to what we originally assumed, we did not find a significant correlation between the intensity and the overall score, which suggests that the perceived intensity of physical activity is not directly related to more or fewer complaints. Similarly, we did not observe a significant relationship between the perceived training intensity and age—another assumption that was rejected by the correlation analysis.

Furthermore, body weight and BMI are strongly correlated (r = 0.71, *p* < 0.01), which is to be expected given that BMI is derived from weight and height. Weight also correlates significantly with height (r = 0.56, *p* < 0.01), indicating that taller individuals naturally tend to have a higher weight. These internal consistencies support the reliability of the data. However, neither BMI nor weight are significantly correlated with the overall score, even though it could be assumed that individuals with a higher BMI might be at greater risk of musculoskeletal complaints.

Regarding the variable age, we could not find any significant correlations with the overall score or perceived intensity. One of the interesting findings here is that age shows a significant negative correlation with the bicycle type, race (r = −0.36, *p* < 0.05), which suggest that younger participants are more likely to use race bikes, while older individuals may prefer other types. This age effect in bike preference might reflect generational trends or considerations regarding physical comfort. However, age does not correlate significantly with BMI or weight in this sample (Table 5).

Finally, looking at the bicycle-type variables, the bicycle type mountain bike shows an interesting pattern, as it is negatively correlated with the overall score (as previously mentioned) and also with BMI (r = −0.35, *p* < 0.05) and bicycle type race (r = −0.45, *p* < 0.01). This indicates that mountain bikers tend to have a lower BMI and are less likely to use race bikes. By contrast, the bicycle type race is negatively associated with height (r = −0.29) and age (r = −0.36, *p* < 0.05), as mentioned earlier. The bicycle type indoor does not show any significant correlations with any of the main variables, which suggests that individuals who train primarily indoors do not differ significantly from others in terms of complaints, BMI or training intensity.

To summarize the findings derived from the correlation analysis, the overall injury score does not strongly correlate with most physical or demographic variables, but it shows a negative correlation with mountain biking. The perceived training intensity correlates strongly with anthropometric measures but not with injury complaints. Some of our basic assumptions such as the relationships between BMI and age, on the one hand, and complaints and injuries, on the other hand, are not statistically supported.

**Figure 1:**
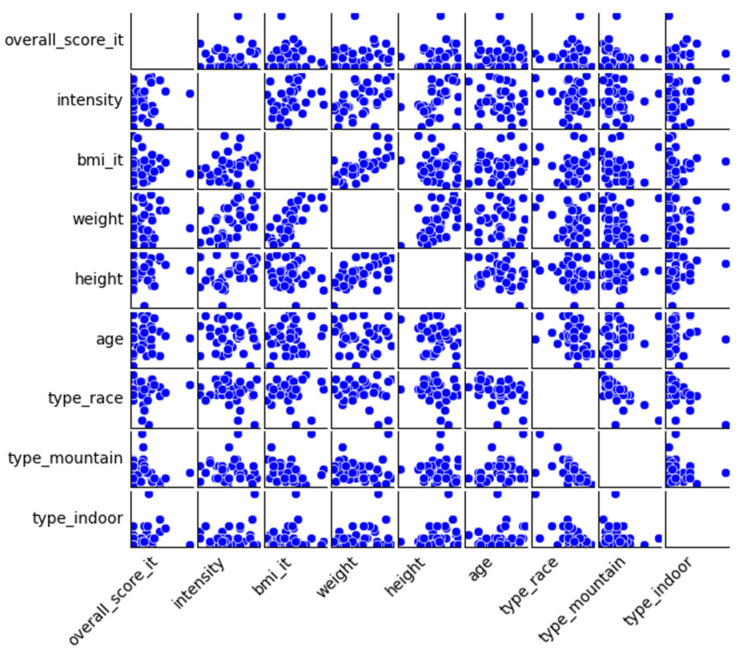
The scatterplot matrix displays the bivariate relationships between the main variables in the correlation matrix (source: own illustration/Python).

The scatterplot matrix in Figure 1 is another way of exploring the bivariate correlations and to identify outliers. As the scatterplots of the overall score show, there are no strong linear relationships between the overall score and the other variables. The distribution of the scores appears to be highly dispersed and random, especially in relation to age, BMI, weight and height. This confirms the statistical results from the correlation analysis of the overall score and its relationship with the independent variables. The scatterplots of the overall scores also indicate that the distribution of the data points is skewed with most participants reporting relatively low scores and only a few higher values.

Whereas the overall score does not show any clear relationship, other pairings in the scatterplot matrix show clearer patterns, one of which is the perceived training intensity that shows a positive relationship with body weight, which aligns with the results of the correlation analysis and suggests that heavier individuals perceive higher intensities or with greater load. To summarize the findings from the inspection of the scatterplot matrix, the matrix visually confirms the results of the correlation analysis. While the overall injury score does not show clear linear correlations, the patterns between physical characteristics and the perceived training intensity remain strong and consistent.

### 5.5. DiD Regression Analysis

In the final step of the statistical analysis, we analyzed the variables through DiD regression models. The section will be subdivided into two sections: the first section analyzes the overall score that represents the general severity and frequency of injuries and complaints. The second section includes the regression model with the perceived training intensity as the dependent variable. In both parts, we tested the independent variables of the correlation analysis, even though they did not show statistically significant correlations in the bivariate correlation matrix. This approach, as we argue, is justified because a lack of significant bivariate correlation does not necessarily imply a lack of predictive value when other variables are controlled for. Moreover, we applied a stepwise regression analysis, starting with a simple DiD regression model without covariates and continuing with multivariate regression models that also include additional controlled variables. We published the best models and excluded those models and variables that decreased the accuracy and robustness of the model.

As we already specified in Section 3, the analysis of the overall injury score and the perceived training intensity consists of four parts. For the first two parts, we focused on the individual time periods, of which the first one starts with the baseline test and ends with the first post-test and the second one starts with the first post-test and ends with the second post-test. In the third part, we analyzed the entire time period from the beginning to the end of the trial; before, in the fourth part, we tested a dynamic DiD regression model that incorporates both time periods with individual treatment effects.

#### 5.5.1. Overall Injury Score as Dependent Variable

The following regression models (models 1–8) include difference-in-differences (DiD) regression models that evaluate the effect of the intervention on the overall level of injury and complaints. The models consist of three main predictors: (1) the time variable, which compares the different time periods, (2) the group assignment, which compares the intervention group with the control group (the intervention group serves as reference group) and (3) the interaction term, which reflects the differential change in the outcome over time between the intervention and control group. This is the basic structure of all static regression models. The interaction term between time and group is the most crucial variable in the model because it estimates the differential change in the outcome between the two groups over time.

In the six static DiD regression models, we analyzed the individual time periods, ranging from the baseline test to the first post-test (models 1–2 shown in Table 6 and Table 7), from the first post-test to the second post-test (models 3–4 shown in Table 8 and Table 9) and from the baseline test to the second post-test (models 5–6 shown in Table 10 and Table 11). For each time period, we published one simple DiD regression model and one extended DiD regression model with additional covariates. As can be seen in the tables below, we found no statistically significant effect of the application of the foam roller on the overall injury score. In model 1, the interaction term suggests that the changes from the baseline test to the first post-test in the intervention group does not significantly differ from the control group with a coefficient of 1.25 and a *p*-value of 0.82. Even if we ignore the insignificance of the interaction term, the coefficient shows a very small effect size, which is likely not empirically relevant. The same is true for the interaction terms in the other seven models, which are far from statistically significant and show very little impact on the overall injury score in terms of effect strength. Moreover, the insignificance of the interaction term does not change even if we account for additional control variables such as BMI, age or the use of bicycle type mountain bike. In other words, in neither of the analyzed time periods could the model confirm our original assumption stating that the application of the Blackroll^®^ results in less severe and frequent health complaints and injuries.

Among the covariates, only the variable bicycle type mountain bike seems to have a statistically significant impact on the overall injury level. For the other covariates, we could not observe any statistically significant effect on the overall injury score. For the usage of mountain bikes, we could estimate a coefficient of −0.22 with a statistical significance of *p* = 0.02, which means that the participants who used mountain bikes (measured in percent of their overall bicycle exercise time), had slightly lower injury scores than the control group. A reduction of 0.22 points per percentage point of mountain bike use suggests a moderate cumulative benefit. In fact, a 20% increase in mountain bike usage would predict a 4.4-point reduction in the overall injury score, indicating a potentially meaningful effect size. As can be seen in the adjusted R-squared, the models also do a poor job in explaining the variance and show a very low model fit. With an adjusted R-squared of 0.076 regression, model 6 which estimated the impact of the intervention with additional covariates from the baseline test to the second post-test turned out to be the model with the highest adjusted R-squared.

Finally, we implemented a dynamic DiD regression model, which incorporates both time periods and accounts for a separate treatment effect for each time-period. The first dynamic model without covariates examines the effects of the intervention on the overall score over time, using only the group assignment (intervention or control) and time indicators. The model explains 6.7% of the variance in the overall score, with an adjusted R-squared of 2.0%, suggesting a very low explanatory power. The treatment effect for the intervention group at the first and second post-tests is represented by the coefficients of 1.25 and 1.27, both of which are not statistically significant (*p* = 0.797 and *p* = 0.793). These coefficients again imply small effect sizes (~10% of baseline) and provide no practical justification for concluding a real treatment benefit. Similar to the previous model, the intervention did not result in a significant change in the overall score at both of the post-test periods compared to the control group (Table 12). Model 8 shows the dynamic regression model with the additional covariates of BMI, age and the use of bicycle type mountain bike. The dynamic treatment effects in Model 8 are captured by the interaction terms P1 and P2, which estimate the additional impact of being in the intervention group at each post-test relative to the control group. Both coefficients are small (−1.24 and −1.29) and clearly non-significant with *p*-values greater than 0.7. These slightly negative coefficients suggest very small and statistically unreliable treatment effects in the opposite direction of what was hypothesized (Table 13).

In sum, neither the static nor the dynamic DiD models provide evidence of a significant impact of the intervention on the overall score across various time periods. Furthermore, the models’ explanatory power is consistently limited with very low adjusted R-squared. In other words, we are not able to confirm our basic assumption in our main hypothesis H1 that the intervention has an impact on the overall level of injury. Although the direction of the time effect and the group differences are in line with our basic assumptions, none of the central variables associated with the intervention reach statistical significance. The only noteworthy predictor is the type of bicycle, which appears to be linked to lower injury scores.

#### 5.5.2. Perceived Intensity as Dependent Variable

In the second part of the DiD regression analysis, we investigated the impact of the intervention on the perceived training intensity. The training intensity represents a scale between 0 and 10 with higher values standing for a more intense training. Similar to the analysis of the intervention on the overall injury score, we tested six static models (model 9–14 shown in Table 14, Table 15, Table 16, Table 17, Table 18 and Table 19) and two dynamic models (models 15 and 16 shown in Table 20 and Table 21) and conducted the same steps as in the previous section.

The intensity increased in both groups over time, but the change in intensity of the intervention group was lower than in the control group. The control group tends to have a lower intensity, but this gap widens over time, as shown by the interaction term. A similar result could be observed in most of the other models as well. In total, five of the ten interaction terms show an interaction term at a significance level of *p* < 0.01 and three interaction at a significance level of *p* < 0.05. Only in the two models, which analyzed the time period from the first post-test to the second post-test, the treatment effect did not reveal a statistically significant result, even after we included additional covariates (model 11 and 12 shown in Table 16 and Table 17).

As the results in the regression tables below show, one of the strongest models in terms of its robustness is model 14 in Table 19, which analyzes the impact of the intervention over the entire trial. The model fits the data quite well as the adjusted R-squared value of 0.886 suggests. The time variable shows that the intensity in both groups increased over time, with a coefficient of 2.145 and high statistical significance (*p* < 0.01), which means that the dependent variable training intensity is on average 2.15 units higher compared to the baseline test—a result that can be explained by the fact that we steadily increased the objective training intensity over time. Most importantly, the interaction term shows a coefficient of 1.35 and a high statistical significance (*p* < 0.05), which means the changes from the baseline test to the first post-test were on average 1.35 units higher in the control group than in the intervention group, suggesting a moderately sized differential effect between the groups—a non-trivial gap given the scale.

For the multivariate models, we decided to prioritize the variable weight as covariate because the variable had the highest correlation with the perceived training intensity, as shown in Section 5.4. Furthermore, the variables of age and bicycle type mountain bike were excluded from the model because of a variety of reasons. First, none of those two variables showed any sign of association with the perceived training intensity in the correlation analysis, even though we expected a correlation, at least between age and training intensity. Second, the inclusion of the two variables significantly reduces the accuracy and fitness of the regression model. Other variables, which we excluded from the model, were the variables BMI and height, even though we found a moderate positive statistically significant correlation between these two variables and the perceived training intensity. The reason for this is that the independent variables in a regression model should not correlate with each other due to the problem of multicollinearity.

Regarding the covariates, the coefficient for body weight is positive and statistically significant in three of the four multivariate regression models, indicating that for each additional kilogram of body weight, while small in magnitude, this association suggests that heavier individuals report a slightly higher perceived intensity—a pattern consistent with higher physical exertion. This effect is relatively small in absolute terms, but it is statistically significant in three of the four multivariate models, which means we can reject the null hypothesis that weight has no impact on intensity. Individuals with a higher body weight tend to perceive their training as more intense, which could reflect differences in physical exertion or perceived effort due to body composition.

In the regression models 15 and 16 shown in Table 20 and Table 21, we conducted dynamic DiD regression models, which includes interaction terms for both time periods. Here, all four interaction terms show effect strengths, ranging from a coefficient of 0.803 to 1.350, two of them with a statistical significance at the *p* < 0.01 level and two at the *p* < 0.05 level. In contrast to the static models, the dynamic models also revealed a statistically significant effect strength for the period from the first post-test to the second post-test.

While the regression models that analyzed perceived training intensity (H2) showed a statistically significant reduction in RPE values in the intervention group, it is important to acknowledge the limitations of self-reported measurements. The RPE scale, which is widely validated and commonly used in sports science, remains subjective and may be influenced by participants’ expectations, motivation or the perceived desirability of improvement. While these potential sources of bias do not entirely invalidate the findings, they do suggest that the reduction in RPE should be interpreted with caution and ideally verified through complementary physiological or performance-based metrics in future studies.

## 6. Discussion

In general, the descriptive analysis showed some interesting insights. A relatively high number of individuals in both groups reported spinal health issues—a fact consistent with various studies indicating the negative health effects of cycling on the spine [3,49,50]. The knee is another body part that has been associated with a significant number of health complaints. A total of 34.3% of participants reported knee problems before the start of the intervention, and both groups reported the same number of knee-related complaints. Like the spine complaints, this result is also consistent with the academic literature, in which knee problems are often considered as one of the most common negative health outcomes among cyclists [49,51].

The primary study goal was to examine the effects of a training-accompanied myofascial self-massage using the Blackroll^®^ on injury occurrence and perceived training intensity within the context of a six-month trial period. Our hypotheses covered both direct treatment effects of the intervention and potential associations with physical and training-related control variables. To analyze the first main dependent variable injury level, we used an overall injury score consisting of the occurrence of an injury at a specific body part, their frequency, timing and intensity. The second main dependent variable perceived training intensity was measured through a BORG scale ranging from 0 to 10.

First of all, our statistical analysis could not confirm the association formulated in H_1_, which assumed that the application of the Blackroll^®^ results in a lower overall injury score. Although some trends could be observed in our descriptive analysis, the changes in the overall score were not robust or consistent enough in our DiD regression models to support the claim that the intervention may improve the injury outcome. The overall injury score decreased during the trial, but the reduction in injury level was observed in both the intervention as well as the control group—one of our findings that highlights the importance of comparing the intervention group with a control group. Thus, H_1_ cannot be confirmed based on the available data. On the one hand, these findings contradict various theoretical considerations and empirical studies that were able to demonstrate an impact of foam rollers on the reduction in pain, injuries or overuse syndromes, such as those reported in [1]. On the other hand, these findings are consistent with studies that did not find a causal relationship between the use of foam rollers and reduced injury levels, for instance, as in [18]. One possible reason why our study could not detect a linkage between foam rollers and a reduction in the overall injury level may be the small sample size with only 35 participants, which increases the risk of Type II errors and may fail to detect an effect that actually exists. Moreover, injuries were measured through self-reported questionnaires, which can lead to biases due to different interpretations of what defines an injury or its severity. Another possible explanation would be that foam rolling may require a longer period of consistent application to show meaningful effects on injury prevention. One central goal for the future could be to improve measurement methods and questionnaires to reduce biases and other errors.

Whereas H_1_ could not be confirmed, the DiD regression analysis provided some evidence in support for H_2_, which proposed that the myofascial self-massage may reduce the perceived training intensity. Although the perceived training intensity increased in both groups, which can be explained by our training protocol with increasing intensity levels throughout the training blocks, participants in the intervention group showed a smaller increase in the perceived training intensity and exertion than those in the control group—a result with relatively high statistical significance. In total, we ran eight DiD regression models to analyze perceived training intensity with ten interaction terms (six static DiD regression models plus two dynamic models, each of the dynamic models include two interaction terms). Before correction four, the models showed a statistically significant effect for the interaction term at the significance level of *p* < 0.01, four additional interaction terms were significant at *p* < 0.05 and two interaction terms showed no statistical significance. To further evaluate the robustness of the findings and to control for FMER, we conducted a Holm–Bonferroni correction on the *p*-values of the interaction terms, which adjusts for multiple comparisons and reduces the likelihood of Type I errors, thereby improving the reliability of the models. The error correction left us with four interaction terms, which were statistically significant after correction, while four interaction terms lost their statistical significance (see Supplementary Materials Table S2).

In addition to the robustness tests and the correction of the *p*-values, we also conducted a Shapiro–Wilk test to assess the normality of residuals for each of the eight regression models that estimated the impact of the intervention on the perceived training intensity. The results indicate that for seven out of the eight models, the residuals did not significantly deviate from a normal distribution with *p*-values above the 0.05 threshold. This supports the assumption of normally distributed residuals for these models, which is an important condition for the validity of linear regression. Only model 12, which estimated the impact of the intervention between the first post-test to the second post-test, showed a significant deviation from normality, suggesting potential concerns with the distribution of residuals in that specific case. Overall, the results indicate that the assumption of normality was generally met across the models (see Supplementary Materials Table S3).

These findings are consistent with [24], which reported that foam rollers reduced the perceived exertion and fatigue among recreational athletes, or with [25], in which researchers found evidence that foam rollers may reduce exertion and fatigue. Although our statistical results are consistent across multiple regression models and various empirical studies, the findings regarding the perceived training intensity should be interpreted with caution. Training intensity was measured using the RPE scale, which relies on participants’ subjective self-assessment rather than objectively measured biomedical indicators. One potential goal for future researchers could be to develop and analyze objective criteria and indicators that reflect the perceived training intensity and exertion.

Regarding the control variables, H_3_, which tested the role of age in the overall injury level and the perceived intensity, could only be partially confirmed. In the dynamic DiD model, age showed a slight impact on the overall injury score, which was statistically significant at the level *p* < 0.05, but this result was not consistent across all models. Neither did the age variable correlate with the perceived training intensity and exertion, which is an unexpected result that contradicts previous studies analyzing the impact of age on injury level or perceived training intensity (see [32,33]). Here too, a small sample size could potentially hide an effect that is potentially present in the broader population. Other reasons include potential selection biases, a limited age variance or other moderating factors.

Similar to the age variable, H_4_, which tested the impact of BMI and weight on the target variables, could only be partially confirmed. Regarding the overall injury score, the models showed no evidence to support the claim that the BMI or weight had an impact on the injury level—a result that contradicts various studies in the academic literature such as in [32,33,34], which found BMI- or weight-related differences regarding injury occurrence in sports. Similar to age, a lack of impact of the BMI on injury level could be partially explained by the small sample size, selection biases or low variation in BMI. Turning to the perceived training intensity, we could observe a correlation between BMI and weight, on the one hand, and the perceived intensity, on the other, which confirms research studies showing an impact of weight and/or BMI on perceived training intensity and exertion, such as in [37]. Within the context of the regression analysis, we dropped the BMI as covariate and included the variable weight in our models because the BMI also correlates with weight, and we wanted to avoid multicollinearity. Although the effect of weight on perceived intensity was statistically significant at a level *p* < 0.05, the effect strength was weak.

Another interesting outcome of our analysis is the influence of the bicycle type on the overall injury level. Originally, we hypothesized that different sports disciplines and subdisciplines may have variations in the frequency and type of injury because they often differ in their physical requirements, environments and risk factors. As the correlation and regression analysis suggest, the usage of mountain bikes showed a small to moderate correlation with a lower overall injury level. One possible theoretical explanation for this could be that mountain bikes generally involve a different seating position and, therefore, may prevent negative outcomes for the spine. Variations between different cycling sub-disciplines could be an interesting research question for future studies.

From a methodological point view, DiD models can provide valuable insights into the effects of interventions or treatments over time. Thus, DiD regression models are considered a useful tool to analyze randomized controlled trials, which account for temporal changes as well as treatment effects. In other words, DiD regression models allow us to analyze causal impacts, while holding additional covariates constant. However, this study may be limited due to multiple reasons. One of these problems is the reliance on self-reported data for the injury symptoms and perceived intensity. We are not able to completely rule out problems caused by self-reporting and subjective self-measurement, which might explain why we could not find statistical evidence for H_1_. Furthermore, a sample size of 35 participants limits the generalizability of our findings and constrains the detection of smaller effect sizes or interaction effects. Another limitation, which should be mentioned at this point, is that the trial took only six months, which means our study can hardly draw conclusions about long-term effects. Possible practical applications for the future could include specifically developed training programs for female and male cyclists of different age groups, performance levels and in various cycling disciplines using the fascia roller after and/or before cycling training as well as alternative endurance training. It would also be worth considering the targeted increased use of the fascia roll for specific complaints.

## 7. Conclusions

This study aimed to verify the long-term-effects of using the Blackroll^®^ for myofascial self-massage during training on health complaints, symptoms of overload and training compatibility in recreational cyclists over a six-month trial period. No evidence was found to suggest that myofascial self-massage reduces injury levels during training, as there was no statistically significant effect of the intervention on the overall injury score. However, we demonstrated that the intervention may reduce perceived training intensity: the intervention group showed a significantly smaller increase in perceived training intensity than the control group. Another interesting outcome was the influence of bicycle type: using mountain bikes was moderately correlated with a lower overall injury level. Further investigation of these findings and their long-term implications for cycling injury prevention is needed. This should include studies with larger sample sizes and follow-up periods.

## Figures and Tables

**Table 1:** Structure of the training blocks at 4 weeks (source: own illustration).

Training Number	Total Number of Intervals	Interval Times (Seconds)	RPM	Intensity
1	10	180	60–65	GA2
		180	90–100	GA1
2	4 + 6	210	65–70	GA2
		210	90–95	GA1
		180	60–65	GA2
		180	95–100	GA1
3	5 + 5	210	65–70	GA2
		210	90–95	GA1
		180	60–65	GA2
		180	95–100	GA1
4	5 + 4	210	65–70	GA2
		210	90–95	GA1
		210	60–65	GA2
		210	95–100	GA1
5	4 + 4	210	65–70	GA2
		210	90–95	GA1
		240	60–65	GA2
		240	95–100	GA1
6	4 + 4	240	65–70	GA2
		240	90–95	GA1
		240	60–65	GA2
		240	95–100	GA1
7	4 + 3	240	65–70	GA2
		240	90–95	GA1
		300	60–65	GA2
		300	95–100	GA1
8	3 + 3	300	65–70	GA2
		300	90–95	GA1
		300	60–65	GA2
		300	95–100	GA1

**Table 2:** Most important variables and structure of dataset (source: own illustration).

Variable	Description
Group	Categorical variable with the attributes *intervention* or *control group*
Age group	Interval-scaled variable with four age groups: *20–30, 31–40, 41–50* and *51–60 years*
Body weight	Metric variable measured in kg
Body height	Metric variable measured in cm
BMI	Body Mass Index is calculated by weight in kg divided by the square of the height in m.
Compensatory training	Semi-metric variable, frequency of the compensatory training with the attributes *once, twice, three* or *more* times a week or *none*.
Cycle type racing bicycle	Metric variable in % relative to other types
Cycle type mountain bike	Metric variable in % relative to other types
Cycle type indoor	Metric variable in % relative to other types
Cycle type other	Metric variable in % relative to other types
Complaint frequency *	Categorical variable with the attributes *rarely* and *frequently*
Complaint type *	Categorical variable with the attributes *pain, spasms, numbness, skin problems, tension, formication, stiffness*, multiple answers were possible
Complaint time *	Nominal scaled variable with the attributes *during cycling*, *after cycling training* or *both*
Complaint intensity *	Ordinal scaled variable with the attributes *tolerable, disturbing* and *intolerable*
Perceived training intensity	Ordinal scaled variable with a scale from 0 to 10 with 10 being the highest perceived training intensity

* The variable frequency, type, time and intensity were measured for several body parts, which are not displayed in the table, including fingers, wrists, arms, shoulders, neck and cervical spine, lower back and lumbar spine, gluteal, hip joint, upper legs, knee, feet and toes or other complaints.

**Table 3:** The total number of complaints and share of individuals per group, who reported a particular complaint and injury for each body part. Complaints are ranked by their total number in the sample. Complaints in this table were measured during the baseline test before the intervention started (source: own illustration/SPSS).

	Baseline Test	
Body Part	Intervention Group	Control Group
Neck/cervical spine	44.4% (8)	23.5% (4)
Knee	33.3% (6)	35.3% (6)
Lumbar spine	22.2% (4)	29.4% (5)
Finger	22.2% (4)	17.6% (3)
Wrists	16.6% (3)	23.5% (4)
Upper legs	16.6% (3)	17.6% (3)
Gluteal	16.6% (3)	11.8% (2)
Shoulders	11.1% (2)	17.6% (3)
Hip joint	16.6% (3)	5.9% (1)
Feet/toes	11.1% (2)	11.8% (2)
Other complaints	5.5% (1)	17.6% (3)
Arms	5.5% (1)	5.9% (1)
Total number of complaints	40	37

**Table 4:** The table shows the sums of the scores for each body part, measured during *baseline test* at the first post-test and second post-test. The numbers in the brackets represent the changes in the scores compared to the previous test (source: own illustration/SPSS).

	Baseline Test		First Post-Test		Second Post-Test	
Body Part	Intervention Group	Control Group	Intervention Group	Control Group	Intervention Group	Control Group
Neck/cervical spine	45	20	15 (−30)	13 (−7)	15 (0)	14 (+1)
Knee	32	33	22 (−10)	27 (−6)	20 (−2)	25 (−2)
Lumbar spine	28	23	23 (−5)	5 (−18)	21 (−2)	6 (+1)
Gluteal	19	18	20 (+1)	12 (−6)	15 (−5)	10 (−2)
Finger	20	16	11 (−9)	10 (−6)	11 (0)	10 (0)
Wrists	15	20	0 (−15)	15 (−5)	0 (0)	13 (−2)
Upper legs	16	13	9 (−7)	5 (−8)	9 (0)	5 (0)
Sholders	11	15	0 (−11)	9 (−6)	0 (0)	8 (−1)
Hip joint	21	3	18 (−3)	0 (−3)	17 (−1)	0 (0)
Feet/toes	8	9	0 (−8)	9 (0)	0 (0)	9 (0)
Other complaints	4	13	0 (−4)	0 (−13)	0 (0)	0 (0)
Arms	4	4	0 (−4)	4 (0)	0 (0)	0 (−4)
Sum/Average change per participant	223	187	118 (−105) −5.83	109 (−78) −4.6	108 (−10) −0.56	100 (−9) −0.53

**Table 5:** The table displays the Spearman correlations between the main variables in the dataset. Correlations with a significance level of *p* < 0.05 are marked with * and correlations with a significance level of *p* < 0.01 are marked with ** (source: own illustration/SPSS).

Variable	Overall Score	Intensity	BMI	Weight	Height	Age	Type Race	Type Mountain	Type Indoor
Overall Score	1.00								
Intensity	0.08	1.00							
BMI	0.13	0.37 *	1.00						
Weight	0.17	0.71 **	0.71 **	1.00					
Height	0.22	0.45 **	−0.10	0.56 **	1.00				
Age	0.06	−0.11	0.10	−0.10	−0.25	1.00			
Type race	0.10	−0.08	0.23	0.02	−0.29	−0.36 *	1.00		
Type mountain	−0.37 *	−0.12	−0.35 *	−0.27	0.02	0.29	−0.45 **	1.00	
Type indoor	0.27	0.03	−0.04	0.13	0.28	0.19	−0.21	0.17	1.00

**Table 6:** Simple linear regression model 1 estimates the impact of the intervention on the overall score from the baseline test to the first post-test (source: own illustration/Python).

Variable	Coefficient	Std. Error	t-Value	*p*-Value	95% Confidence Interval
Intercept	12.39	2.64	4.69	0.00	[7.11, 17.67]
Time	−5.83	3.74	−1.56	0.12	[−13.3, 1.632]
Group (control group)	−1.39	3.79	−0.37	0.72	[−8.96, 6.19]
Interaction term	1.25	5.37	0.23	0.82	[−9.47, 11.96]
R^2^: 0.06|R^2^-adjusted: 0.01					

**Table 7:** Regression model 2 estimates the impact of the intervention and additional covariates from the baseline test to the first post-test on the overall score (source: own illustration/Python).

Variable	Coefficient	Std. Error	t-Value	*p*-Value	95% Confidence Interval
Intercept	10.83	16.78	0.65	0.52	[−22.71, 44.36]
Time	−5.8	3.65	−1.59	0.12	[−13.09, 1.5]
Group (control group)	−4.21	3.92	−1.08	0.29	[−12.05, 3.62]
Interaction term	1.23	5.23	0.24	0.82	[−9.23, 11.69]
BMI	−0.26	0.68	−0.38	0.71	[−1.62, 1.12]
Age	0.32	0.19	1.67	0.1	[−0.07, 0.71]
Type mountain bike	−0.22	0.09	−2.39	0.02	[−0.4, −0.04]
R^2^: 0.143|R^2^-adjusted: 0.061					

**Table 8:** Simple linear regression model 3 estimates the impact of the intervention on the overall score from the first post-test to the second post-test (source: own illustration/Python).

Variable	Coefficient	Std. Error	t-Value	*p*-Value	95% Confidence Interval
Intercept	6.56	1.82	3.61	0.00	[2.93, 10.18]
Time	−0.56	2.57	−0.22	0.83	[−5.68, 4.57]
Group (control group)	−0.14	2.61	−0.06	0.96	[−5.35, 5.06]
Interaction term	0.03	3.69	0.01	0.99	[−7.33, 7.38]
R^2^: 0.001|R^2^-adjusted: -0.044					

**Table 9:** Regression model 4 estimates the impact of the intervention and additional covariates from the first post-test to the second post-test on the overall score (source: own illustration/Python).

Variable	Coefficient	Std. Error	t-Value	*p*-Value	95% Confidence Interval
Intercept	−8.98	12.12	−0.74	0.46	[−33.2, 15.25]
Time	−0.4	2.51	−0.16	0.88	[−5.42, 4.63]
Group (control group)	−1.46	2.71	−0.54	0.59	[−6.87, 3.95]
Interaction term	−0.11	3.60	−0.03	0.98	[−7.30, 7.09]
BMI	0.42	0.50	0.83	0.41	[−0.58, 1.42]
Age	0.20	0.14	1.48	0.14	[−0.07, 0.47]
Type mountain bike	−0.11	0.06	−1.77	0.08	[−0.24, 0.02]
R^2^: 0.092|R^2^-adjusted: 0.005					

**Table 10:** Simple linear regression model 5 estimates the impact of the intervention on the overall score from the baseline test to the second post-test (source: own illustration/Python).

Variable	Coefficient	Std. Error	t-Value	*p*-Value	95% Confidence Interval
Intercept	12.39	2.89	4.79	0.00	[7.23, 17.56]
Time	−6.39	3.66	−1.75	0.085	[−13.69, 0.91]
Group (control group)	−1.39	3.71	−0.37	0.71	[−8.8, 6.02]
Interaction term	1.27	5.25	0.242	0.81	[−9.20, 11.75]
R^2^: 0.07|R^2^-adjusted: 0.03					

**Table 11:** Regression model 6 estimates the impact of the intervention and additional covariates from the baseline test to the second post-test on the overall score (source: own illustration/Python).

Variable	Coefficient	Std. Error	t-Value	*p*-Value	95% Confidence Interval
Intercept	11.53	16.04	0.72	0.48	[−20.52, 43.58]
Time	−6.45	3.57	−1.81	0.075	[−13.58, 0.68]
Group (control group)	−4.17	3.83	−0.37	0.71	[−8.8, 6.02]
Interaction term	1.34	5.12	0.26	0.79	[−8.88, 11.57]
BMI	−0.28	0.66	−0.42	0.68	[−1.59, 1.04]
Age	0.32	0.19	1.66	0.1	[−0.07, 0.7]
Type mountain bike	−0.22	0.1	−2.42	0.02	[−0.4, −0.04]
R^2^: 0.156|R^2^-adjusted: 0.076					

**Table 12:** Regression model 7 shows a dynamic regression model and estimates the impact of the intervention and additional covariates from the baseline test to the first post-test and from the first post-test to the second post-test on the overall score (source: own illustration/Python).

Variable	Coefficient	Std. Error	t-Value	*p*-Value	95% Confidence Interval
Intercept	12.39	2.38	5.21	0.00	[7.67, 17.11]
Time (P1)	−4.59	3.46	−1.33	0.19	[−11.46, 2.28]
Time (P2)	−5.12	3.46	−1.48	0.14	[−11.99, 1.75]
Group (control group)	−1.39	3.41	−0.41	0.69	[−8.16, 5.38]
Interaction term (P1)	1.25	4.83	−0.26	0.80	[−10.82, 8.33]
Interaction term (P2)	1.27	4.83	−0.26	0.79	[−10.85, 8.31]
R^2^: 0.067|R^2^-adjusted: 0.02					

**Table 13:** Regression model 8 shows a dynamic regression model and estimates the impact of the intervention and additional covariates from the baseline test to the first post-test and from the first post-test to the second post-test on the overall score (source: own illustration/Python).

Variable	Coefficient	Std. Error	t-Value	*p*-Value	95% Confidence Interval
Intercept	6.88	12.42	0.55	0.58	[−17.78, 31.53]
Time (P1)	−4.58	3.36	−1.36	0.18	[−11.26, 2.09]
Time (P2)	−5.12	3.36	−1.52	0.13	[−11.79, 1.56]
Group (control group)	−3.73	3.45	−1.08	0.28	[−10.57, 3.12]
Interaction term (P1)	−1.24	4.69	−0.27	0.79	[−10.55, 8.07]
Interaction term (P2)	−1.29	4.69	−0.27	0.78	[−10.60, 8.02]
BMI	−0.06	0.51	−0.12	0.91	[−1.07, 0.95]
Age	0.28	0.14	1.97	0.05	[−0.00, 0.57]
Type mountain bike	−0.18	0.07	−2.73	0.01	[−0.32, −0.05]
R^2^: 0.147|R^2^-adjusted: 0.075					

**Table 14:** Regression model 9 estimates the impact of the intervention from the baseline test to the first post-test on the perceived training intensity (source: own illustration/Python).

Variable	Coefficient	Std. Error	t-Value	*p*-Value	95% Confidence Interval
Intercept	3.964	0.196	20.194	0.000	[3.572, 4.356]
Time	2.145	0.278	7.726	0.000	[1.591,2.699]
Group (control group)	−0.892	0.282	−3.165	0.002	[−1.454, −0.329]
Interaction term	0.803	0.398	2.015	0.048	[0.007, 1.598]
R^2^: 0.723|R^2^-adjusted: 0.710					

**Table 15:** Regression model 10 estimates the impact of the intervention from the baseline test to the first post-test on the perceived training intensity with additional covariates (source: own illustration/Python).

Variable	Coefficient	Std. Error	t-Value	*p*-Value	95% Confidence Interval
Intercept	1.378	1.008	1.367	0.176	[−0.636, 3.392]
Time	2.145	0.266	8.059	0.000	[1.613, 2.677]
Group (control group)	−0.708	0.279	−2.537	0.014	[−1.265, −0.151]
Interaction term	0.803	0.382	2.015	0.039	[0.040, 1.565]
Weight	0.033	0.012	2.611	0.011	[0.008, 0.057]
R^2^: 0.749|R^2^-adjusted: 0.734					

**Table 16:** Regression model 11 estimates the impact of the intervention from the first post-test to the second post-test on the perceived training intensity (source: own illustration/Python).

Variable	Coefficient	Std. Error	t-Value	*p*-Value	95% Confidence Interval
Intercept	6.109	0.176	34.76	0.000	[5.758, 6.460]
Time	0.809	0.249	3.256	0.002	[0.313, 1.306]
Group (control group)	−0.089	0.252	−0.352	0.726	[−0.592, −0.415]
Interaction term	0.548	0.357	1.535	0.130	[−0.165, 1.260]
R^2^: 0.376|R^2^-adjusted: 0.348					

**Table 17:** Regression model 12 estimates the impact of the intervention from the first post-test to the second post-test on the perceived training intensity with additional covariates (source: own illustration/Python).

Variable	Coefficient	Std. Error	t-Value	*p*-Value	95% Confidence Interval
Intercept	4.891	0.936	5.223	0.000	[3.021, 6.761]
Time	0.809	0.247	3.275	0.002	[0.316, 1.303]
Group (control group)	−0.002	0.259	−0.009	0.993	[−0.520, 0.515]
Interaction term	0.548	0.355	1.544	0.127	[−0.161, 1.256]
Weight	0.015	0.012	1.324	0.190	[0.008, 0.038]
R^2^: 0.393|R^2^-adjusted: 0.355					

**Table 18:** Regression model 13 estimates the impact of the intervention from the baseline test to the second post-test on the perceived training intensity (source: own illustration/Python).

Variable	Coefficient	Std. Error	t-Value	*p*-Value	95% Confidence Interval
Intercept	3.964	0.161	24.571	0.000	[3.642, 4.287]
Time	2.954	0.228	12.948	0.000	[2.499, 3.410]
Group (control group)	−0.891	0.232	−3.851	0.000	[−1.354, −0.429]
Interaction term	1.350	0.327	4.124	0.000	[0.697, 2.004]
R^2^: 0.885|R^2^-adjusted: 0.879					

**Table 19:** Regression model 14 estimates the impact of the intervention from the baseline test to the second post-test on the perceived training intensity with additional covariates (source: own illustration/Python).

Variable	Coefficient	Std. Error	t-Value	*p*-Value	95% Confidence Interval
Intercept	2.158	0.841	2.567	0.013	[0.479, 3.836]
Time	2.954	0.222	13.314	0.000	[2.511, 3.398]
Group (control group)	−0.763	0.233	−3.281	0.002	[−1.228, −0.299]
Interaction term	1.350	0.318	4.241	0.000	[0.714, 1.986]
Weight	0.023	0.010	2.188	0.032	[0.002, 0.044]
R^2^: 0.892|R^2^-adjusted: 0.886					

**Table 20:** Regression model 15 shows a dynamic regression model and estimates the impact of the intervention on the intensity from the baseline test to the first post-test and from the first post-test to the second post-test (source: own illustration/Python).

Variable	Coefficient	Std. Error	t-Value	*p*-Value	95% Confidence Interval
Intercept	3.964	0.178	22.223	0.000	[3.610, 4.318]
Time (P1)	2.145	0.252	8.502	0.000	[1.644, 2.646]
Time (P2)	2.954	0.252	11.711	0.000	[2.454, 3.455]
Group (control group)	−0.892	0.256	−3.483	0.001	[−1.399, −0.384]
Interaction term (P1)	0.803	0.362	2.217	0.029	[0.084, 1.521]
Interaction term (P2)	1.350	0.362	−3.730	0.000	[0.632, 2.069]
R^2^: 0.815|R^2^-adjusted: 0.805					

**Table 21:** Regression model 16 shows a dynamic regression model and estimates the impact of the intervention and additional covariates from the baseline test to the first post-test and from the first post-test to the second post-test on the perceived intensity (source: own illustration/Python).

Variable	Coefficient	Std. Error	t-Value	*p*-Value	95% Confidence Interval
Intercept	2.094	0.767	2.730	0.008	[0.572, 3.616]
Time (P1)	2.145	0.246	8.726	0.000	[1.657, 2.633]
Time (P2)	2.954	0.246	12.018	0.000	[2.467, 3.442]
Group (control group)	−0.759	0.255	−2.976	0.004	[−1.265, −0.253]
Interaction term (P1)	0.803	0.353	2.276	0.025	[0.103, 1.503]
Interaction term (P2)	1.350	0.353	−3.828	0.000	[0.650, 2.050]
Weight	0.024	0.009	2.504	0.014	[0.005, 0.042]
R^2^: 0.826|R^2^-adjusted: 0.815					

## Data Availability

The data are available on request to the corresponding author.

## Supplementary Materials

The following supporting information can be downloaded at: https://www.mdpi.com/article/10.3390/healthcare13111337/s1, Figure S1 S15: histogram age group intervention and control group (cited in the manuscript on pp 15); Figure S2 S15: histogram BMI intervention and control group (cited in the manuscript on pp 15); Table S1 S9: Time schedule for the data collection process during the preparation and intervention; Table S2 S28 Holm-Bonferroni correction applied to the *p*-values of the interaction terms in the models; Table S3 S28 Shapiro-Wilk test to assess the normality of the residuals.
