# Supplementary material for: Long-Term-Effects of Training-Accompanied Myofascial Self-Massage on Health Complaints, Symptoms of Overload, and Training Compatibility in Recreational Cyclists
Source: Healthcare (Basel). 2025 Jun 4;13(11):1337. doi: 10.3390/healthcare13111337 (PMC12155503; doi:10.3390/healthcare13111337)
Supplement: Supplementary file 1 [file healthcare-13-01337-s001.zip › healthcare-3630897-Supplementary Figures.pdf]

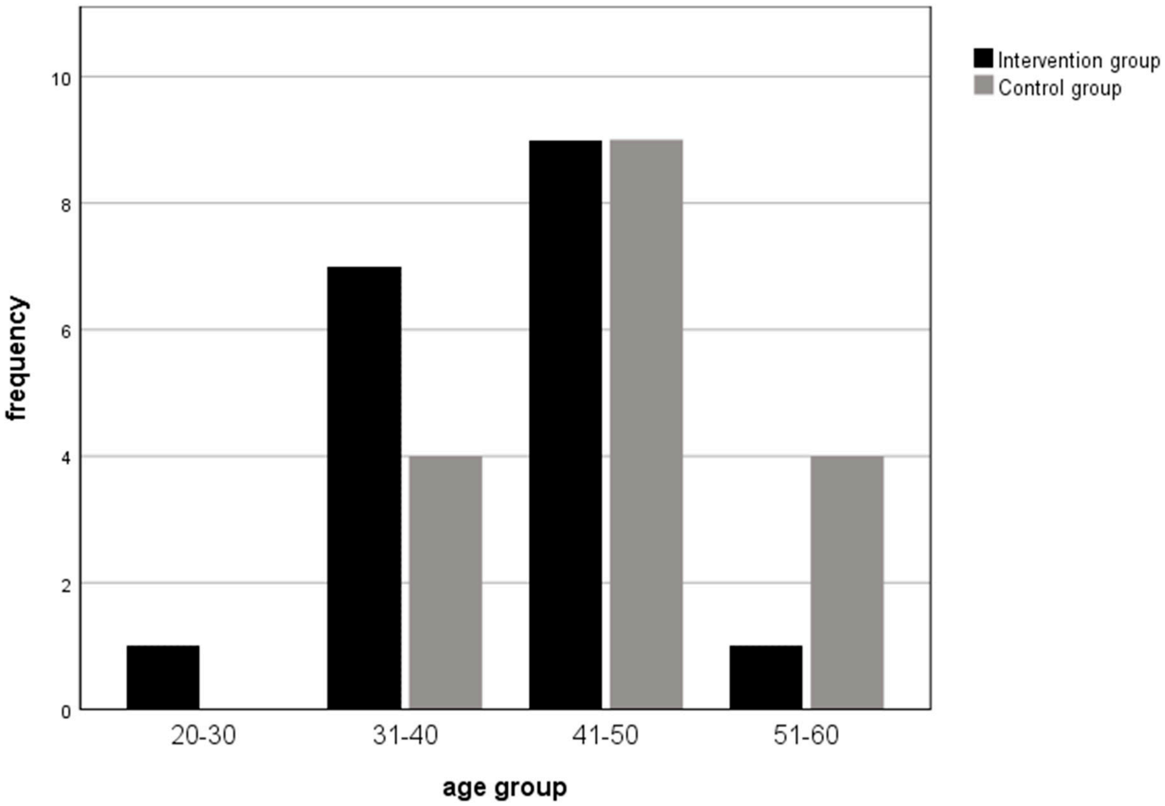

Figure S1. S15 Histogram Age Group Intervention und Control Group.own source.SPSS

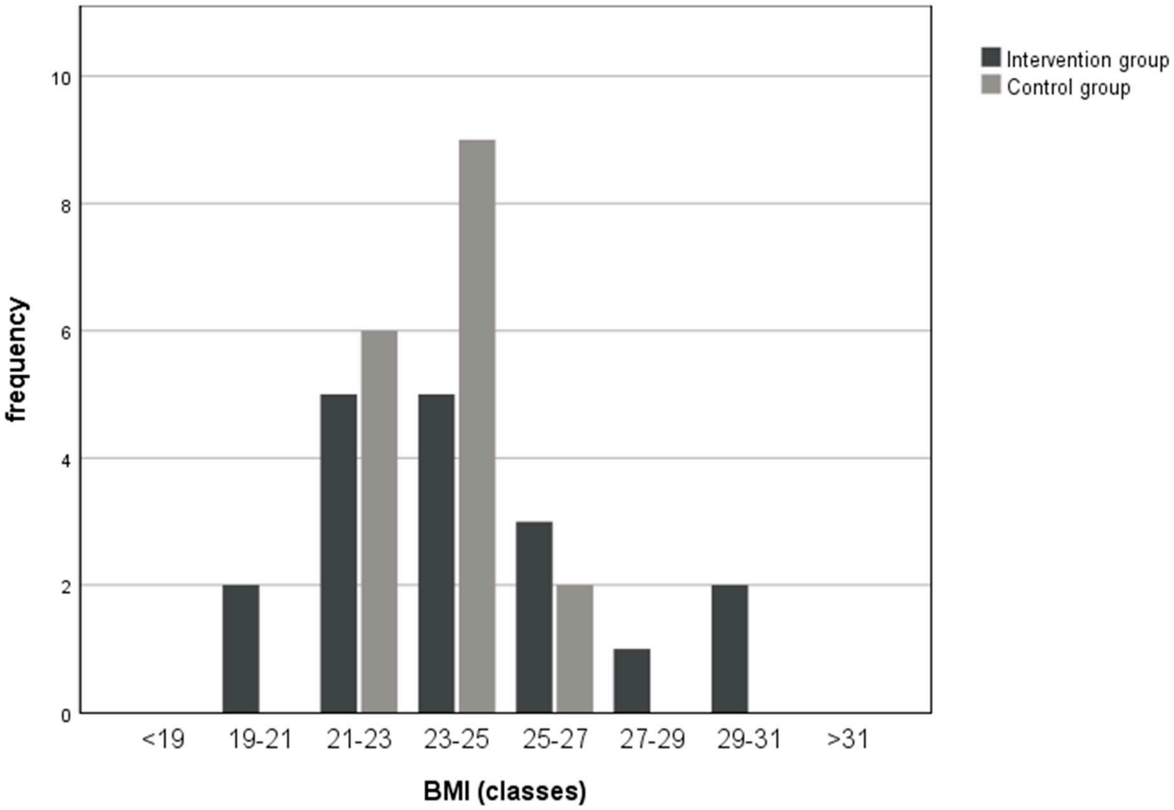

Figure S2. S15 Histogram BMI Intervention und Control Group.own source.SPSS
