# Supplementary material for: Long-Term-Effects of Training-Accompanied Myofascial Self-Massage on Health Complaints, Symptoms of Overload, and Training Compatibility in Recreational Cyclists
Source: Healthcare (Basel). 2025 Jun 4;13(11):1337. doi: 10.3390/healthcare13111337 (PMC12155503; doi:10.3390/healthcare13111337)
Supplement: Supplementary file 1 [file healthcare-13-01337-s001.zip › healthcare-3630897-Supplementary Table S1.pdf]

Table S1. S9: Time schedule for the data collection process during the preparation and intervention phases (Source: own illustration)

Month/Week	Milestones and data collection
March-September (week 12-38)	Recruitment of participants
September (week 39)	Detailed instructions for participants
September (week 40)	Pretests
October (week 41-42)	Baseline tests
October (week 43)	Beginning of intervention phase
January (week 1)	1st post-test for intervention group
January (week 2)	1st post-test for control group
March (week 13)	2nd post-test for intervention group
April (week 14-15)	2nd post-test for control group
April (week 15)	End of intervention phase
