# Supplementary material for: Long-Term-Effects of Training-Accompanied Myofascial Self-Massage on Health Complaints, Symptoms of Overload, and Training Compatibility in Recreational Cyclists
Source: Healthcare (Basel). 2025 Jun 4;13(11):1337. doi: 10.3390/healthcare13111337 (PMC12155503; doi:10.3390/healthcare13111337)
Supplement: Supplementary file 1 [file healthcare-13-01337-s001.zip › healthcare-3630897-Supplementary Table S2.pdf]

**Table S2.** S28: Holm-Bonferroni correction applied to the p-values of the interaction terms in the models analyzing the impact of the intervention on the perceived training intensity (Source: own illustration/Python)

| Original p-values | Holm-Bonferroni p | Significant ($p < 0.05$) |
|---|---|---|
| 0.048 | 0.156 | False |
| 0.039 | 0.125 | False |
| 0.130 | 0.254 | False |
| 0.127 | 0.254 | False |
| 0.000 | 0.000 | True |
| 0.000 | 0.000 | True |
| 0.029 | 0.125 | False |
| 0.000 | 0.000 | True |
| 0.025 | 0.125 | False |
| 0.000 | 0.000 | True |
