# Supplementary material for: Long-Term-Effects of Training-Accompanied Myofascial Self-Massage on Health Complaints, Symptoms of Overload, and Training Compatibility in Recreational Cyclists
Source: Healthcare (Basel). 2025 Jun 4;13(11):1337. doi: 10.3390/healthcare13111337 (PMC12155503; doi:10.3390/healthcare13111337)
Supplement: Supplementary file 1 [file healthcare-13-01337-s001.zip › healthcare-3630897-Supplementary Table S3.pdf]

Table S3. S28: Shapiro-Wilk test to assess the normality of the residuals (Source: own illustration/Python)

Model	W-statistic	p-value	Normal (p > 0.05)
Model 9	0.9795	0.3069	True
Model 10	0.974	0.1514	True
Model 11	0.9715	0.1175	True
Model 12	0.9599	0.0235	False
Model 13	0.9889	0.7975	True
Model 14	0.9863	0.6463	True
Model 15	0.9867	0.3823	True
Model 16	0.978	0.0771	True
